# Head and Neck Squamous Cell Carcinoma in Elderly Patients: Role of Radiotherapy and Chemotherapy

**DOI:** 10.3390/cancers14030472

**Published:** 2022-01-18

**Authors:** Morena Fasano, Ida D’Onofrio, Maria Paola Belfiore, Antonio Angrisani, Valentina Caliendo, Carminia Maria Della Corte, Mario Pirozzi, Sergio Facchini, Marianna Caterino, Cesare Guida, Valerio Nardone, Alfonso Reginelli, Salvatore Cappabianca

**Affiliations:** 1 Department of Precision Medicine, Faculty of Medicine and Surgery, University of Campania “L. Vanvitelli”, 80138 Naples, Italy; morena.fasano@unicampania.it (M.F.); mariapaola.belfiore@unicampania.it (M.P.B.); angrisani.antonio.4@gmail.com (A.A.); valentina.caliendo@unicampania.it (V.C.); carminiamaria.dellacorte@unicampania.it (C.M.D.C.); Mario.pirozzi@unicampania.it (M.P.); Sergio.facchini@unicampania.it (S.F.); Marianna.caterino@unicampania.it (M.C.); alfonso.reginelli@unicampania.it (A.R.); Salvatore.cappabianca@unicampania.it (S.C.); 2 Unit of Radiation Oncology, Ospedale del Mare, ASL Napoli 1 Centro, 80147 Naples, Italy; ida.donofrio@aslnapoli1centro.it (I.D.); cesare.guida@aslnapoli1centro.it (C.G.)

**Keywords:** HNSCC, radiotherapy, elderly, chemotherapy

## Abstract

**Simple Summary:**

The focus of this review deals with the management of elderly patients with head and neck squamous cell carcinoma, discussing the role of clinical management, geriatric evaluation and therapeutic approaches (radiation therapy and systemic therapies).

**Abstract:**

Head and neck squamous cell carcinomas (HNSCC) constitute the sixth most common malignancy worldwide, with approximately 25–40% of the diagnosed patients older than 70 years. HNSCC patients are often frail and frequently have multiple comorbidities due to their unhealthy lifestyle, and evidence suggests that older patients may receive less aggressive and suboptimal treatment than younger patients with the same disease status. The aim of this review is to depict and summarize the evidence regarding the different strategies that can be used in the clinical management of elderly HNSCC patients. Key references were derived from a PubMed query. Hand searching and clinicaltrials.gov were also used. This paper contains a narrative report and a critical discussion of clinical approaches in the context of elderly HNSCC.

## 1. Introduction

Head and neck squamous cell carcinomas (HNSCC) constitute the sixth most common malignancy worldwide, representing approximately 6% of all new cancer cases. Although the majority of HNC occurs during the fifth and sixth decades of life, approximately 25–40% of the patients are over 70 years old [1]. The elderly is the fastest-growing segment of the population of Western countries. Despite an increasing number of human papilloma virus (HPV)-related cancers that particularly affect young patients, most HNSCC still occur mainly in patients over the age of 50. In the choice of treatment, it must be considered that the “chronological” age does not always coincide with the “biological” age. Generally, patients with head HNSCC are often frail and frequently have multiple comorbidities due to their unhealthy lifestyle [2]. In addition, there is evidence that older patients with HNSCC may receive less aggressive and suboptimal treatment than younger patients with the same disease status [1]. For this reason, in the choice of treatment, it is important to evaluate some characteristics both related to the patient (performance status, comorbidity or symptoms related to the disease, life expectancy, compliance to treatment), and features related to the tumor (TNM stage, tumor grading, debulking or curative strategies) as age may not affect the tolerability profile of treatment and denying standard therapy to elderly patients only on the basis of age may not be entirely justified. The use of concurrent chemoradiotherapy (CCRT) for HNSCC has been shown to be effective and safe in older cancer patients. A meta-analysis by Pignon et al. showed an improvement in overall survival of 4.5% at 5 years and an absolute benefit for concurrent CCRT of 6.5% compared to radiotherapy alone; however, this benefit was only in patients under the age of 70 [3,4,5]. However, it is unclear whether older patients might really benefit from more aggressive therapies. Age-related biological changes cause a decline in multiple physiological functions. For this reason, decision making on treatment in older patients requires multidisciplinary evaluation and risk assessment. Older patients are potentially at increased risk for treatment-related toxicity due to changes in pharmacokinetics and pharmacodynamics resulting from physiological changes in age [1]. Extended surgery is offered less frequently to older patients, and non-surgical monomodal treatment, preferably radiotherapy, is often preferred. In order to prevent treatment-related morbidity or mortality in this population, many patients may be at risk of undertreatment. Therefore, giving up appropriate and standard curative treatment often leads to a deterioration of the prognosis and quality of life of these patients. Certainly, decisions on the treatment of elderly patients with HNSCC are often influenced by the challenge of predicting their outcomes with respect to treatment tolerance, complications, quality of life, and survival. Therefore, it is evident that finding a balance between intensive treatment and therapeutic nihilism is essential in elderly patients [2].

## 2. The Role of Geriatric Assessment

It has long been known that unresectable patients have a greater benefit from CCRT than radiotherapy alone. Thus, combined treatment should be preferred. Starting from this assumption, in the context of multidisciplinarity, geriatric evaluations are important to discriminate whether an elderly patient can perform a combined treatment. A multidisciplinary, multidimensional process has been defined and proposed to provide a guiding tool to drive treatment decisions in oncology with a comprehensive approach. Such a tool, named comprehensive geriatric assessment (CGA), examines several domains, including functional status, comorbidity, cognition, mental health status, fatigue, social status and support, nutrition, and presence of geriatric syndromes [6]. The use of CGA in older cancer patients may help to differentiate fit or unfit patients to define who could tolerate a more aggressive treatment approach [1], as it identifies patients with a higher risk of chemotherapy-related adverse events [7]. Furthermore, GA parameters indicating health problems have been related to worse survival, higher risk of hospitalization and postoperative morbidity [8]. Non-oncologic GA-guided interventions are also common and feasible in daily practice: these include physical therapy referrals, nutritional care, psychiatric and neuropsychological consultations, social support assistance [9,10]. The role of CGA remains controversial, and there is still no validation in the HNSCC setting: preliminary results of a study with CGA in HNSCC patients showed that CGA changed treatment decisions in 8% of patients [11,12], but as of now, there is no evidence it improves results in HNSCC patients. Nevertheless, according to many experts, all patients should undertake the CGA before treatment. However, systematic adoption of the CGA is referred to as cumbersome and time consuming. In response, several shorter tools have been developed to identify high-risk patients who may benefit from further geriatric evaluation: the one commonly used is the G8, whose results well correlate with CGA findings [13]. G8 includes only eight items: age, decreased oral intake, weight loss in the past 3 months, mobility, neuropsychological problems, BMI, ≥3 medications daily, and self-reported overall health. Nevertheless, the ELAN-ONCOVAL trial, published in 2019, endorsed the use of the EGE (ELAN geriatric evaluation) in lieu of the G8 screening tool, which was considered not appropriate for elderly HNSCC patients [11,12]. In the locally advanced (LA) HNSCC setting, small studies using GA have been conducted. Both Wanderwalde et al. [14] and Pottel et al. [15] have found that although QoL was generally reduced during treatment, those with a suitable baseline IADL recovered after therapy, while QoL continued to decline in those with baseline impairments. Altered baseline G8 in Neve et al. [16] has been linked to worse postoperative outcomes and lower treatment completion in the RT/CRT arm. An Italian survey by AIRO on elderly HNSCC patients showed that while the majority of cases were discussed in a multidisciplinary setting, a geriatrician was rarely part of the MDT, and comprehensive geriatric assessment was performed in only 10% of radiotherapy-oncology departments, thus risking under or overtreatment due to wrong frailty assessments [17]. There is still the need for a larger, more HNSCC-focused study that may be used in the future as the basis for risk stratification and decision making, as there is now available only limited evidence to support default geriatric assessment. As Dickstein analysis showed, treatment interruptions in the elderly were associated with worse OS but not disease-related outcomes; therefore, it can be inferred that interruptions may be multifactorial and mostly dependent on comorbidities, socioeconomic status, age, etc. [18]. Finally, other parameters could be included in the CGA, such as the inclusion of immune parameters (neutrophil-to-lymphocyte ratio, C-reactive protein to albumin ratio, and so on) [19,20]. In this regard, more recently, Zhou et al. introduced a novel clinical signature to predict survival of elderly patients with oral squamous cell carcinoma that included hemoglobin level, BMI, and NLR [21].

## 3. Treatment Choice

LA-HNSCC is frequently treatable, but multimodal treatment, which includes a combination of surgery, radiation, and/or chemotherapy, is often required to achieve an ambitious level of disease cure. Older patients frequently present with cardiovascular or pulmonary comorbidities and a poor nutritional status [13]. Current literature recommendations do not provide clear answers on this. Most studies include younger patients, with the older population underrepresented, and inclusion criteria generally limit participation to 65–70 years of age. The elderly population is often undertreated, as age is an independent risk factor for less intense and lower than average treatment rates [22] even though it has been repeatedly confirmed that medical assessment is paramount and that age alone must not guide treatment decisions. Moreover, even the various meta-analyses do not agree on the definition of elderly patients, as some consider the elderly patient to be over 65 years old, others the patient to be over 70 years old. This does not allow to have clear and comparable data on the efficacy and tolerability of multimodal treatment.

### 3.1. Radiotherapy

Radiotherapy is a mainstay of the management of head and neck tumors, especially in the LA setting, both alone and combined with chemotherapy. Among the few insights into the efficacy and the toxicity (both acute and late) of radiotherapy in older patients, the relevant analysis carried out by Pignon et al. on five prospective EORTC trials of HNSCC patients receiving radiotherapy concluded that prognosis was not determined or influenced by age as local control and overall survival comparisons between age groups showed no statistically significant differences, and despite the unavailability of a full assessment of late toxicity in these cohorts of patients, the objective toxicity profile reported was also similar when comparing patients older than 65 with younger, though similar grades of toxicity resulted having a larger impact on the older groups [23]. Selection bias within the studies cannot be neglected, as the population presented with suitable performance status and thus resulted in a similar OS regardless of age. Moreover, those trials enrolled patients younger than 75 years [2]. A more recent retrospective analysis suggested that both radiotherapy and chemoradiotherapy are realistic opportunities in selected aged patients with suitable performance status. Both treatments showed high locoregional control rates in this setting while performing worse than in the younger cohorts: median OS was 34 months, compared to around 72 months in the EORTC 22931 trial [24] or the 44.9 months in the RTOG 9501 trial [25]. The toxicity profile was unfavorable with 56.1% acute CTCAE grade 3/4 toxicities and chronic toxicities less serious but widespread, with performance status and age found to be important prognostic factors, likely due to the multiple comorbidities and median life expectancy. It was underlined that since performance status analysis is highly subjective with high interobserver variability, it would benefit from an objective geriatric assessment [26]. To date, the vast majority of RCTs did not enroll aged nor frail patients, providing information and useful results, which may be inferred only to a proportion of HNC patients (median age of diagnosis of HNC is 60 years, but this is only true for 70% of new diagnoses). Patients over 70 years old represent an important proportion (nearly 30%) of HNC (*), and in the upcoming years, the incidence of HNC is expected to increase by 64% in the elderly [27,28]. With the exception of selected older adults with optimal performance status and absence of impaired functionality due to comorbidities, the intrinsic features of vulnerable, frail, aged patients with LA-HNSCC do not leave room for intensive treatment (radical surgery, chemoradiation); therefore, radiotherapy alone remains the only viable option, in the light of the evolution in radiotherapy techniques (e.g., IMRT/VMAT) which allowed to a significant reduction in adverse effects [29]. Given the unfavorable prognosis in this subset of patients [30], the ultimate treatment goal aims to obtain an effective objective response and local control, therefore impacting on QoL rather than prolonging survival with a high risk of toxicity, whereas the OS gain could be less relevant in the light of a short baseline life expectancy due to age and comorbidities. Despite the struggles carried by physicians and experts in HNC on the optimal therapeutic strategy for older adults with intermediate PS, low agreement has been reached, and multiple radiotherapy schedules have been developed in the last two decades. The large meta-analysis of 63 trials involving 10,741 patients “MACH-NC” provided robust evidence for indication of concurrent chemoradiation in HNC patients younger than 70 years; conversely, effects in older patients showed no benefit, at times with detrimental results. A low proportion of older adults were enrolled (accounting for approximately 4%) in the RCTs evaluated; therefore, firm conclusions on concomitant CRT in the elderly were not available [31]. Bourhis et al. [32] conducted a deep meta-analysis of radiotherapy in squamous cell carcinomas of head and neck (MARCH), concluding that a hyper fractionated schedule, as well as accelerated radiotherapy, led to better locoregional control (6.4% at 5 years; *p* < 0.0001), which was particularly efficient in reducing local failure, whereas the benefit on nodal control was less pronounced. Unfortunately, the benefit was not significant for patients over 70 years old (OR 1.08, 95%CI: 0.89–1.30). Furthermore, hypofractionationated and split-course schedules result in a reduced number of accesses to the hospital and a radiobiological benefit in terms of lower acute toxicity.

On the other hand, concerns of hypofractionation regard the possible detrimental effect in terms of tumor control and late toxicity, but the probability of registering late adverse events in this subset of patients is low given the ‘a priori’ life expectancy; it is mandatory to deliver a radical total dose with the intent to ensure adequate local control. Due to the above considerations, hypofractionation has been widely adopted in the treatment of this subset of patients. Other strategies included the choice of even higher doses for a few fractions (stereotactic body radiotherapy, SBRT) or the choice of palliative radiotherapy for patients with poor performance status.

We report in Table 1 and Table 2 a summary of the reviewed studies’ results and toxicity.

#### 3.1.1. Hypofractionated and Normofractionated Radiotherapy

A widely adopted strategy in clinical practice to treat elderly HNC patients in western European countries is the prescription of a first course of hypofractionated radiotherapy with non-curative doses, possibly followed by a second course, in selected patients with a partial response and suitable tolerance, a few weeks later. Crucial information regarding the efficacy of split-course hypofractionated RT in non-metastatic frail HNC patients is warmly expected from the ELAN-RT trial (NCT01864850). Bledsoe et al. [33] reported the results of the unconventional accelerated split course of hypofractionated radiotherapy in patients with anticipated intolerance to traditional CRT. In their eight years’ experience, 65 patients were consecutively treated and evaluated; among them, 39 had no distant metastasis or recurrent tumors. Adopting a regimen of 60 to 72 Gy delivered in two courses of 30–36 Gy in 10 fractions of 2 Gy each, with an interval of 3 to 5 weeks to allow recovery from symptoms, tumor response was seen in more than 90% of cases and a surprisingly high median LFS of 25 months was reported. The median OS was 8.9 months, in accordance with similar studies previously published; however, important intervention bias is to be noticed as some early patients were treated with 3D-RT while others received IMRT. The authors conceptualized the importance of LRC over survival in frail HNC patients, accounting for the impact of worsening morbidity due to these aggressive neoplasms. 

Benhmida et al. [28] already reported solid results of IMRT mainly delivered in two courses of 30 Gray (Gy)/10 fraction (fr), 5 days a week, separated by a rest of 2 to 4 weeks midcourse or using a slightly different schedule involving simultaneous integrated boost of 44 Gy (20 daily fractions of 2.2 Gy) on the PTV1 (prophylactic volume) and 55 Gy (20 fractions of 2.75 Gy) on the PTV2 (focused on the primitive tumor and involved nodes), with a rest of 2 weeks midcourse as indicated in the ELAN-RT trial protocol. A total of 75 patients were enrolled, showing a 1y- and 2y-LRC of 72.8% and 51.7%, respectively, a median OS of 19.3 months, acceptable acute toxicity, and only 3 cases of G ≥3 late toxicity according to CTCAE v.5. Previously adopted hypofractionated regimens for advanced HNC in vulnerable patients such as the french IHF2SQ [37] showed suitable results with an improved median OS (12.9 months), although patient characteristics were heterogeneous, and the nature of the study was retrospective. The objective response rate, evaluated 2 months after completion of radiotherapy, reached 54% for the primary tumor, with 11 patients (14.5%) achieving a complete response and 30 patients (39.5%) a partial response. Strategies to treating older adults with HNC through a normofractionated scheme are still being investigated, tailoring and possibly reducing volumes of treatment instead of the total radiation dose or number of fractions, and in this direction, we are waiting for ongoing studies such as the SINGLE-ARM, NON-RANDOMIZED, MULTICENTER STUDY on non-elective vulnerable elderly radiotherapy “NEVER” (NCT04832555).

**Figure 1:**
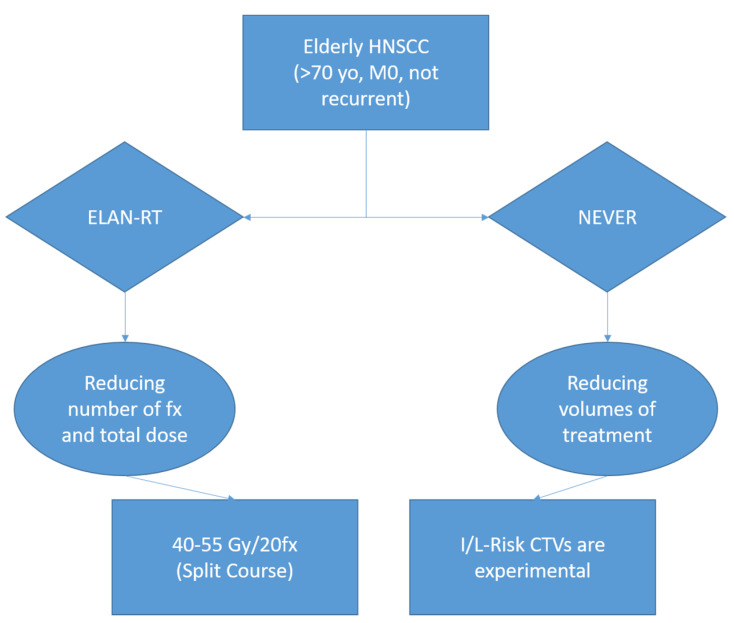
Opposite strategies in ongoing studies for elderly HNSCC. HNSCC = head and neck squamous cell carcinoma, yo: years old, RT = radiotherapy, Gy = Gray, I/L = intermediate/low-risk target volumes, CTV = clinical target volume. Inclusion criteria between studies widely overlap, although some slight differences exist between the two studies. Therefore, we invite the reader to consult Clinicaltrial.gov for further information regarding ELAN-RT (NCT01864850) and NEVER (NCT04832555) protocols.

The two opposed RT strategies in ongoing studies on vulnerable elderly are resumed in Figure 1.

#### 3.1.2. SBRT

Thanks to advances in radiotherapy techniques, newer strategies have been developed using stereotactic body radiation therapy (SBRT), initially as an ultimate strategy to re-irradiate recurrences but as a primary treatment for the elderly and medically inoperable patients with HNC. An interesting retrospective experience recently reported 1y-LRC of 73% and 1y-OS of 64%, with a low toxicity rate (CTCAE G3 = 3%) in a cohort of 66 patients older than 80 and unfit for surgical or conventional radiation treatment for HNC, receiving SBRT with a total dose of 35–40 GY to GTV and 30 Gy to the clinical target volume in bi-weekly five fractions, between 2011 and 2016 [35]. The biologically equivalent doses reached 59.5–72 Gy, accounting alpha/beta ratio of 10, and it is worth noting the low toxicity rate of this approach, with only 3% of the patients showing G3 toxicity and no patients showing G4. A larger retrospective Canadian study enrolled 114 patients (median age of 81 y) and reported successful, feasible, and effective use of SBRT for a total dose of 40–50 Gy in 5 fractions. Although the more heterogeneous sample (including 69 patients with distant metastasis, recurrent tumors, or non-HNC primary tumors) of this study, we considered substantial the results regarding the median LRC of 23.4 months for previously untreated HNC primaries, despite slightly higher toxicity, probably due to the irradiation of elective volumes (25 Gy to PTV low risk) [34].

#### 3.1.3. Palliative Radiotherapy

Among the relevant studies regarding optimal palliation RT, the evaluation of objective response, outcomes, and toxicity profile of the adoption of the “Christie scheme” performed by Al Mamgani et al. on more than 150 advanced HNC patients demonstrated an impressive ORR (73% complete or partial response) [40]. The scheme proposed a total dose of 50 Gy in 16 fractions (3125 Gy/Fr) five or four fractions per week in a time span of four weeks; the median OS was 17 months, which dropped to 10 months in patients with major comorbidities, substantially better than average survival of untreated advanced stage HNC previously estimated being 100 days on average [41]. The above-mentioned treatment scheme was associated with a high rate of toxicity (a feeding tube was needed in 65% of cases, and in 45% of the whole cohort, G3 or higher dysphagia was experienced during the RT treatment) while QoL was retrospectively assessed only in 12 patients one year after RT ended, in a subgroup with a median age of 66 years.

Positive experiences were described using the “QUAD-SHOT” schedule to optimize palliative RT in HNC with modern techniques, underlying the value of such a strategy, which was first developed by the RTOG 8502 in the 1980s, and subsequently adapted for HNC [39], which consisted of 2 days of twice-daily fractionation with a fraction size of 3.7 Gy (14.8 Gy per cycle) repeated at 3- to 6-week intervals for a total of three cycles with an RT dose of 44.4 Gy. In the above-mentioned phase II trial, ORR and overall treatment tolerance achieved suitable results with a suitable safety profile (G3 toxicity in only 5% of the patients, no G4 toxicity), although the median local control was limited with a PFS of 3.1 months. Similarly, the repeated short course accelerated radiation therapy (SHARON) was tested in this subset of patients, with promising preliminary results [38]. A very recent Japanese report on the use of the afore-mentioned scheme with a more modern approach in terms of RT delivery such as VMAT, IGRT, and ART showed in small series of HNC patients no acute G3 or higher toxicity [42].

Finally, Bonomo et al. [36] explored the use of the accelerated hypofractionated radiotherapy (AHRT) for a total dose of 40Gy in 15 fractions with modern RT techniques (3D-RT, IMRT) in medically unfit patients (G8 score < 14, with the 89% of the patients showing a G8 < 11). The trial showed an ORR of 66.6%, assessed at 2 months, with 36% of the patients showing G3 toxicity, but the local control at 1 year was unsatisfactory (23%). Moreover, the review and meta-analysis carried out by Desideri et al. provided an excellent overview of palliative RT in elderly HNC patients [43].

### 3.2. Chemoradiotherapy

One of the main analyses of the role of CRT for both adjuvant and definitive treatment in older HNSCC patients comes from a metanalysis by Pignon et al. published in 2009 [44], including 93 trials and 17,346 patients. Adding chemotherapy to radiotherapy showed improvements regardless of the primary site and mostly for the stages III–IV LA concurrent setting. However, the benefit of chemotherapy was age dependent, with the best results in the younger than 50 years of age population and no survival benefit in the over 71 population. It must be noted that the population over 71 was very small compared to the rest of the participants and that difference in survival may also be due to other causes of non-cancer-related death, thus making it troubling to observe any benefit due to dilution effect.

This meta-analysis, one of the most well-known sources of evidence about CRT in the elderly population, included data from trials from 1965 to 2000. Over the last few decades, there has been a decrease in tobacco and alcohol-related HNSCC (often accompanied by more comorbidities) with an increase in HPV related OPSCC even in the older population, thus making the general population significantly different from the participants in the historical trials; moreover, newer radiotherapy techniques allowed for a decrease in toxicity rates [13]. Hence, it is possible that these radical changes in population and treatment techniques may positively influence the efficacy index in the modern elderly population.

More recently, the meta-analysis has been updated, including 115 randomized, controlled trials for a total of 28,978 included patients. The updated results suggest that further intensifying chemoradiotherapy, using hyperfractionated radiotherapy with concomitant chemotherapy (HFCRT) or induction chemotherapy with taxane, cisplatin, and fluorouracil followed by concomitant chemotherapy ICTaxPF-CLRT, could improve outcomes over chemoradiotherapy for the treatment of locally advanced head and neck cancer [4] in selected patients with few comorbidities and suitable performance status. Therefore, its role in the elderly population is disputed despite its efficacy.

Concurrent CRT regimens recommended by most guidelines contemplate platinum-based regimens, mainly using cisplatin. RT alone is less effective than platinum-based CRT, as demonstrated by Pignon metanalysis [44] and multiple succeeding RCT [45,46].

A set of clinical criteria was established to define platinum fit or unfit patients, which could be used routinely in clinical practice. Absolute contraindications are PS, renal dysfunction, otologic or neurologic disorders, HIV/AIDS with CD4 count <200/uL, pregnancy, and lactation, known hypersensitivity to platinum-based therapy. Age, comorbidities, borderline otologic, neurologic or renal function, immunocompromised conditions, previous platinum therapy, nutritional status characterize a high-risk patient group for which the use of cisplatin is dependent on physician’s choice [47].

High-dose cisplatin is borne by significant toxicities (nausea and vomiting, myelosuppression, nephrotoxicity, ototoxicity, mucositis, etc.), and older, frail patients are often unfit to cisplatin due to renal, cardiac, or other comorbidities. Due to unacceptable toxicities, about 40% of patients do not complete all three planned cycles of high-dose cisplatin: nevertheless, a cumulative dose above 200 mg/m^2^ seems enough to produce a therapeutic effect [48].

In fact, a weekly cisplatin regimen was investigated as a substitute for the classic 100 mg/m^2^ three-weekly cisplatin, with pharmacological investigations demonstrating that toxicity may be dose dependent [49]. Weekly cisplatin was found to be noninferior to high-dose cisplatin [50], with no significant difference in overall survival and/or response rate, both in the postoperative and in the definitive setting. Weekly cisplatin was indeed better tolerated, with fewer grade 3–4 adverse events, especially in terms of myelotoxicity, nausea, and vomit, whereas dysphagia and weight loss rates may be comparable or worse with weekly cisplatin [48].

Carboplatin has been a frequent replacement due to the similar mode of action and lower rates of toxicity [51]. There are no direct comparison studies between the two platinum-based regimens in HNSCC. One noninferiority trial in the NPC population suggests equivalent efficacy with better tolerability for the carboplatin arm [52]. Furthermore, a 2015 metanalysis showed no significant difference in the OS and LRC at 3 years and other toxicities between cisplatin and carboplatin, with a higher OS at 5 years for cisplatin-based chemotherapy [53].

In the U.S., CRT use in the >65 years HNSCC population has increased in the last decades, thanks to the rising use of cetuximab plus radiotherapy in the elderly population or those with comorbidity. However, there is only one trial showing benefit from the cetuximab plus RT combination over RT alone in LRC end OS. Most patients enrolled were below 70 years of age without significant comorbidities [54], and indeed following analysis showed the older groups were less likely to benefit from the combination [55]. Cetuximab’s principal benefit is, of course, the reduced toxicity profile, with decreased nephrological, auditory, and hematologic AEs, though skin toxicity is very frequent with concurrent cetuximab plus RT treatment. A retrospective metanalysis showed better OS and DFS with cisplatin compared to cetuximab, significantly regarding LRC (48), whereas two RCT (De-ESCALaTE HPV and RTOG 1016) showed no benefit for cetuximab compared to cisplatin. Early, late, and overall toxicity rates were comparable between cetuximab and cisplatin (albeit its profile was different) with inferior results for tumor control and survival in the cetuximab + radiotherapy arm [56,57,58].

As such, cisplatin-based CRT remains the standard of care but, for frailer population ineligible to cisplatin therapy, different therapeutic approaches in concurrent systemic treatment are available: weekly cisplatin, with similar efficacy results but noninferior toxicity rate and reduced cumulative cisplatin dose in comparison with three-weekly cisplatin; bioradiotherapy with cetuximab, which, while perceived safer, needs careful selection according to the toxicity profile (skin reaction) [59].

## 4. QoL and Development of Predictive Scores

It is intuitive that every procedure mentioned above could negatively impact patients’ quality of life (QoL) [2,60]. Elderly patients are commonly affected by this nosocomial entity; unfortunately: almost a quarter of HNSCC patients are 70 years old or older, but the numbers are bound to increase in the coming future [61,62,63]. Furthermore, their intrinsic frailty, deeply connected to considerable clinical differences between elderly and younger HNSCC patients [64,65], delivers exclusion or underrepresentation in most clinical trials [24,46,54,66,67,68].

Nowadays, QoL assessment has become more prominent as a key-treatment feature, highlighting correlations between its perception and therapeutic outcomes. Bozec et al. aimed to assess the evolution and/or changes in QoL in HNSCC patients undergoing oncologic surgery and to determine possible predictive factors of post-treatment QoL alterations [60]. The median age in this trial was 63.5 ± 10.3 years. The analysis showed no significant deterioration of global QoL and no significant increase in general symptoms between pre- and postoperative periods; in contrast, the impact of treatment was evident for the role and social functioning scales, as well as for most head and neck symptoms. The main predictors were found to be tumor stage T or N, tumor site, and treatment modalities. As discussed in other QoL evaluation studies [69,70,71], there were no significant changes in global QoL and no drastic increase in general symptoms after curative treatment.

It could be explained by the fact that general health status and symptoms were already impaired before treatment. The significant deterioration in the role and social functioning highlights the main consequences of HNSCC treatment: affecting the patients’ appearance and their ability to communicate, as also showed by Veldhuis et al. in a previous study [72], and these impairments could explain the significant increase in financial difficulties reported in HNSCC patients worldwide [60,72,73]. Treatment modalities also demonstrated a remarkable impact on increasing fatigue scores: baseline neurocognitive and activity impairment risk factors (excess of alcohol consumption, smoking), which are common in HNSCC, and undergoing multimodal therapy might negatively and synergically deteriorate QoL of these patients.

Emotional functioning was the most deteriorated activity both before and after the treatment, even though emotional functioning was the only one that tended to improve. Holloway et al. showed that both psychosocial and physiologic factors influenced QoL in patients with HNSCC, but QoL measures were most strongly influenced by psychosocial considerations [74]; moderate or severe preoperative depressive connotations had significantly decreased postoperative functional performance status, increased narcotic use, decreased completion of adjuvant therapy and a longer length of hospital stay [75].

Functional impairment was also assessed, demonstrating that opening mouth, dental status, salivary, speeching, and swallowing functions were the main function affected by the treatment [76], and also the items that had an effect on perceived QoL, as demonstrated by Chaukar et al. [73]; these disorders, however, could be interpreted as long-term side effects of RT, suggesting that adjuvant therapy and not primary surgery could be the main factor affecting patients’ QoL. RT has been reported as the major mouth opening limitation risk factor [77]; altered sense of tastes, restricted diet, and changed physical appearance could all affect social eating and relations, leading to a deterioration in these activities [60,78]. Multidisciplinary management has become mandatory to improve the nutritional and general performance status of patients, highlighting the need for individualized follow-up for head and neck patients. Education level has also been reported to correlate with the use of painkiller drugs and poorer generic and cancer-specific QoL and decline of physical activities [79].

Rühle et al. tried to implement everyday clinical practice with a score that could bring important prognostic information in elderly patients who undergo (chemo)radiation or adjuvant therapy [80]. Their retrospective analysis, conducted on two separate cohorts (development and validation), proved that there was a prognostic correlation between some variables (represented by Karnofsky Performance Score, age, Charlson Comorbidity Index, C reactive protein) and overall survival. Karnofsky Perfomance Score has been widely known for its relevance in patients’ general status assessment in every stage of oncological treatment (pre, during, post intervention); in modern days, it also demonstrated to be an independent prognostic parameter in neoplasms [81,82]. They could then divide their cohort into three separate prognostic populations (favorable, intermediate, unfavorable) based on the final score obtained, thus demonstrating a correlation between each variable and the outcome, as shown in Table 3 and Table 4. The study itself happened to have some flaws (no geriatric assessment performed, retrospective design); it suggested a new way to stratify HNSCC patients, providing a promising new tool in treatment management, nonetheless. An example of the parameters in Table 3 and Table 4 are used in practice to stratify an elderly patient‘s prognostic factor subgroup.

## 5. Conclusions

The older adult with HNSCC still remains a relevant clinical issue: in the past, older age has often been an independent exclusion factor from many randomized controlled trials, and even when data are available, one must keep in mind the important improvements of the last decades, both in the therapeutic approaches (e.g., radiotherapy advancements) and in the holistic assessment of the patient. Nowadays, we know that age alone may not be enough in determining treatment fitness, but there is much more to do before frailty assessment, and geriatric supportive care can be incorporated into everyday clinical practice.

New gero-centric trials to enrich our knowledge are crucial to reach a definite consensus for the treatment of older patients, free from the bias of the past. Personalized treatment according to diagnosis, functional status, and treatment tolerance must be the aim of head and neck clinicians.

Finally, the role of immune checkpoint inhibitors (ICIs) in the clinical management of LA-HNSCC is currently under investigation in several clinical trials in concurrent, neoadjuvant, and adjuvant settings [83,84,85,86]. ICIs are active agents in recurrent and metastatic HNSCC, and their role with RT in the curative setting is yet to be defined [83,87,88,89]. Their profile of safety is very attractive, especially for elderly patients [90,91,92]. In addition, the combination of ICIs with SBRT is an attractive treatment in patients with oligometastatic or oligoprogressive HNSCC to boost the anti-tumor immune response [93,94].

## Figures and Tables

**Table 1:** Characteristics and results of the reported radiotherapy studies.

Authors, Year	Sample Size (Recruitment Period)	Type of Study	Radiotherapy Regimen	Radiotherapy Technique	Radiation Dose Prescribed (Gy/Fr)	Median Local Control (Months)	Median OS (Months)	Number of Drop-Off (%)
**RADICAL RT INTENT—CONVENTIONAL DOSE FRACTIONATION**								
Bledsoe et al., 2016 [33]	65 (2002–2010)	Retrospective	SCAHRT	3D-CRT or IMRT	60–72/33	25	8.9	7 (11%)
Benhmida et al., 2020 [28]	75 (2012–2019)	Retrospective	SCH-RT	IMRT/VMAT	60/20	11.5	19.3	3 (4%)
**RADICAL RT INTENT—SBRT**								
Al-Assaf et al., 2020 [34]	131 (2011–2016)	Retrospective	-	SBRT (IGRT)	40–50/5	23.7 in the untreated HNC primary patients	-	17 (13%)
Gogineni et al., 2019 [35]	66 (2011–2018)	Retrospective	-	SBRT	35–40/5	28.3	-	0
**PALLIATIVE RT INTENT**								
Bonomo et al., 2017 [36]	36 (2011–2016)	Retrospective	AHRT	3D-RT or IMRT/VMAT	40/15	5	12	3 (9%)
Monnier et al., 2013 [37]	78 (1997–2008)	Retrospective	IHF2SQ	2D or 3D-CRT	48/16	10	12.9	12 (15%)
Al Magmani et al., 2009 (33)	154	Prospective/ Retrospective	“Christie Scheme”	2D or 3D	50/16	13	17	0
Ferro et al.,2020 [38]	17 (2010–2018)	Prospective	SHARON	IMRT	40/5	-	-	9 (53%)
Corry et al, 2005 [39]	30 (1999–2003)	Prospective Phase II	“QUAD SHOT”	-	42/12	3.1	5.7	16 (53%)

**Table 2:** Summary of objective response rates and toxicity reported.

Authors, Year	Sample Size (Recruitment Period)	Radiation Dose Prescribed (Gy/Fr)	Objective Response Rate	Acute Toxicity (Scale)	Late Toxicity (Scale)
**RADICAL RT INTENT—CONVENTIONAL DOSE FRACTIONATION**					
Bledsoe et al., 2016 [33]	65 (2002–2010)	SCAHRT 60–72/33	91% PR or CR	G ≥ 4: 0 G3: 42% CTCAE v5	-
Benhmida et al., 2020 [28]	75 (2012–2019)	SCH-RT 60/20	72%?	G ≥ 4: 1% G3: 5% CTCAE v5	G ≥ 4: 0 G3: 4% CTCAE v5
**RADICAL RT INTENT—SBRT**					
Al-Assaf et al., 2020 [34]	131 (2011–2016)	SBRT 40–50/5	84%	G4: 1% G3: 52%	G4: 2%
Gogineni et al., 2019 [35]	66 (2011–2018)	SBRT 35–40/5	-	G ≥ 4: 0 G3: 3% RTOG	G ≥ 3: 0RTOG
**PALLIATIVE RT INTENT**					
Bonomo et al., 2017 [36]	36 (2011–2016)	AHRT 40/15	66%	G ≥ 4: 0 G3: 36% CTCAE v4	-
Monnier et al., 2013 [37]	78 (1997–2008)	IHF2SQ 48/16	54%	G ≥ 4: 0 G3: 6% EORTC-RTOG	G3–4: 12% EORTC-RTOG
Al Mamgani et al., 2009 [40]	154	“Christie Scheme” 50/16	73%	G5: 0 G ≥ 3: 45–65% EORTC-RTOG	G ≥ 4: 4.5% G3: 29% EORTC-RTOG
Ferro et al., 2020 [38]	17 (2010–2018)	SHARON 40/5	88%	G ≥ 3: 0 EORTC-RTOG	G ≥ 3: 0 EORTC-RTOG
Corry et al., 2005 [39]	30 (1999–2003)	“QUAD SHOT” 42/12	53%	G ≥ 3: 0 CTCAE v3	-

Abbreviations: SCAHRT = split-course accelerated hypofractionated radiotherapy; IHF2SQ = French regimen indicating 2 fractions of 3 Gray per day (days 1 and 3), during the first, third, fifth, and seventh week of treatment with concurrent platinum-based chemotherapy; AHRT: accelerated hypofractionated radiotherapy; SCH-RT: split-course hypofractionated radiotherapy; SBRT: stereotactic body radiation therapy; SHARON: short course accelerated radiation therapy consisting of two courses of 20 Gray each delivered 5 Gray/fraction bid for two consecutive days; IMRT: intensity-modulated radiotherapy; VMAT: volumetric arc radiotherapy; IGRT: image-guided radiotherapy; 2D-,3D-RT: 2- or 3-dimensional radiotherapy.

**Table 3:** Total scores and their attribution to prognostic subgroups.

Favorable	Intermediate	Unfavorable
0 pts	0.5–2	2.5

**Table 4:** Variable and points given based on beta regression coefficient of the parameters.

Variable	KPS ≤ 70%	CCI ≥ 6	(CRP)
Points	1	1	0.5
